# Distribution of Airway Findings in ANCA-Associated Vasculitis: A 20-Year Observational Analysis

**DOI:** 10.3390/diagnostics15010074

**Published:** 2024-12-31

**Authors:** Megan M. Sullivan, Maximiliano Diaz Menindez, Hassan Baig, Anushka Irani, Ronald Butendieck, Benjamin Wang, Florentina Berianu, Carolyn Mead-Harvey, Andy Abril, Vikas Majithia

**Affiliations:** 1 Department of Internal Medicine, Division of Rheumatology, Mayo Clinic, Scottsdale, AZ 85259, USA; 2 Department of Internal Medicine, Scottsdale, AZ 85259, USA; diazmenindez.maximiliano@mayo.edu; 3 Department of Internal Medicine, Division of Pulmonology, Mayo Clinic, Jacksonville, FL 32224, USA; baig.hassan@mayo.edu; 4 Department of Internal Medicine, Division of Rheumatology, Mayo Clinic, Jacksonville, FL 32224, USA; irani.anushka@mayo.edu (A.I.); butendieck.ronald@mayo.edu (R.B.); wang.benjamin@mayo.edu (B.W.); berianu.florentina@mayo.edu (F.B.); abril.andy@mayo.edu (A.A.); majithia.vikas@mayo.edu (V.M.); 5 Department of Quantitative Health Sciences, Mayo Clinic, Scottsdale, AZ 85259, USA; mead-harvey.carolyn@mayo.edu

**Keywords:** ANCA vasculitis, granulomatosis with polyangiitis, prognostication, tracheobronchial, subglottic stenosis, latent class analysis

## Abstract

**Objective:** Pulmonary involvement is commonly observed in anti-neutrophil cytoplasmic antibody (ANCA)-associated vasculitis (AAV), presenting with manifestations such as diffuse alveolar hemorrhage, inflammatory infiltrates, pulmonary nodules, and tracheobronchial disease. We aimed to identify distinct subgroups of tracheobronchial disease patterns in patients with anti-neutrophil cytoplasmic antibody (ANCA)-associated vasculitis (AAV) using latent class analysis (LCA), and to evaluate their clinical characteristics and outcomes. **Methods:** We conducted a retrospective cohort study using electronic medical records of patients aged >18 years diagnosed with AAV and tracheobronchial disease between 1 January 2002 and 6 September 2022. Patients with follow-up <6 months were excluded. LCA was employed to identify disease subtypes based on 10 pre-defined indicators. Maximum likelihood estimation with 10 repetitions per model ensured robustness in model selection, guided by the Akaike information criterion (AIC). Patient and disease characteristics were summarized and compared across predicted classes. Statistical analyses included Kruskal–Wallis and Fisher’s exact tests for continuous and categorical variables, respectively. The primary outcome was time to relapse of the tracheobronchial inflammation after starting immunosuppressive medication, analyzed using the Kaplan–Meier method and log-rank tests. Secondary outcomes included severity of pulmonary disease on pulmonary function tests, endoscopic interventions, tracheostomy, or mortality during follow-up. **Results:** Among 136 identified AAV patients assessed for tracheobronchial involvement, 111 (81.6%) were included after excluding 25 without tracheal or bronchial disease. Predominant findings included subglottic stenosis (91.0%), lower tracheal stenosis (16.2%), and bronchial stenosis (17.1%). LCA identified a three-class model as optimal: tracheal predominant (*n* = 94), tracheobronchial (n = 12), and bronchial predominant (n = 5). Tracheal predominant patients showed reduced risk of ear, eye, and lower respiratory manifestations, with milder obstruction on pulmonary function testing (PFT). Tracheobronchial-class patients were prone to saddle nose deformity (50%), extensive lower respiratory involvement (91.7%), and renal disease (66.7%). Bronchial predominant patients exhibited severe obstructive disease (median forced expiratory volume in 1 s (FEV1)% predicted: 58, IQR 34–66; FEV1/forced vital capacity (FVC) ratio: 56.9, interquartile range (IQR) 43–63.3) but lacked systemic AAV manifestations. LCA classes did not predict outcomes such as endoscopic intervention, tracheostomy, recurrent tracheobronchial narrowing, or mortality. **Conclusion:** LCA shows promise in subtype stratification of AAV patients, yet its utility in predicting outcomes and guiding treatment remains limited based on our analysis. Future studies with enhanced phenotypic data and larger cohorts are warranted to improve predictive accuracy.

## 1. Introduction

Anti-neutrophil cytoplasmic antibody (ANCA)-associated vasculitis (AAV) is a group of disorders including granulomatosis with polyangiitis (GPA), microscopic polyangiitis (MPA), and eosinophilic granulomatosis with polyangiitis (EGPA) [1,2]. These illnesses are complicated by small-vessel vascular inflammation that is associated with ANCA antibodies specific for proteinase-3 and myeloperoxidase.

GPA and MPA, and to a lesser degree EGPA, are well known to cause upper and lower respiratory manifestations [3] These can include pulmonary nodules, tracheobronchial involvement, diffuse alveolar hemorrhage, and less commonly, interstitial lung disease or bronchiectasis [4]. These manifestations can cause a variety of symptoms including hoarseness, cough, dyspnea, stridor, hemoptysis, and wheezing with upper airway involvement. Hemoptysis, pleuritic chest pain, and shortness of breath are common with lower airway involvement. Specifically, tracheobronchial disease has been reported in 19.3–23% of patients with AAV. In particular, subglottic stenosis, defined as narrowing of the airway between the vocal cords and the lower cricoid cartilage, is the most common manifestation of tracheobronchial AAV [5,6,7,8,9,10]. These are devastating complications which can lead to upper airway obstruction and potentially life-threatening disease [5,6,7,8,9]. Bronchial involvement occurs in approximately half of those with tracheal disease [7,11]. Despite the frequent upper airway involvement in AAV, there is a dearth of data about its epidemiology, outcomes, and prognosis, with only a few studies providing information about this manifestation.

Identification of patient subsets at particular risk for airway disease is a significant unmet need in AAV. The prevalence of tracheobronchial disease in patients with GPA has been the focus of a recent cohort where subglottic stenosis and endobronchial disease were reported in 10% and 6% of patients with GPA, respectively [7]. Interestingly, the tracheobronchial disease was reported to occur without disease activity in other organs and did not necessarily correlate to disease activity overall. This study suggested that certain epidemiologic characteristics of the patient, such as female sex and younger age of onset, may be associated with the phenotype of tracheobronchial disease and outcomes.

In contrast, a much higher prevalence of upper airway involvement of 70% was reported from a cohort in Iran but the involvement was not well defined [8,12]. In this cohort, upper airway involvement was associated with a higher disease-associated damage and worse outcomes. In a separate cohort from Turkey, ear–nose–throat (ENT) involvement that included bloody nasal discharge, sinusitis, subglottic stenosis, otomastoiditis, or hearing loss was 37%, but the individual prevalence was not defined [13]. ENT involvement was felt to be associated with better outcomes, but no differentiation was made regarding specific manifestations.

Lastly, in a separate cohort from Australia, the prevalence of tracheobronchial disease manifesting as subglottic and main bronchial airway stenosis was described in 17.1% of patients with GPA [14]. Many patients required multiple therapeutic endobronchial interventions to alleviate symptomatic airway obstruction. This report also highlighted the clinical dilemma that the stenotic lesion may result from ongoing active inflammatory disease or be related to exaggerated post-inflammatory cartilaginous fibrosis and structuring. It is unclear if these are amenable to immunosuppressive treatment with recent reports showing benefit with immunosuppressive therapy, particularly rituximab, as maintenance therapy [15,16].

The existence of specific subgroups within ANCA-positive patients experiencing tracheobronchial involvement that could guide prognostication and therapy remains unknown. Determining these phenotypic groups can prognosticate the outcomes and improve management decisions. LCA is a method used to differentiate potential groups and has been applied in similar instances [5,10]. LCA is considered superior to standard statistical methods for utilizing complex data to evaluate for “latent”, or hidden subgroups that can be clinically relevant. Gu et al. utilized LCA to differentiate radiographic manifestations of microscopic polyangiitis, proposing a four-class model (“infiltrative”, “fibrotic”, “bronchiectatic”, and “nonspecific”) that correlated with disease manifestations, relapse rates, and cumulative survival [5]. Survival rates at one year were lowest for the infiltrative (51.9%) and fibrotic (62.5%) classes compared with the nonspecific (74.0%) and bronchiectatic (85.7%) classes. However, their analysis did not include tracheobronchial disease as a variable.

LCA has also been utilized in relapsing polychondritis, another disease process that can affect the tracheal and bronchial airways and is akin to ANCA vasculitis [17]. In this evaluation of 73 patients, LCA identified three subgroups which correlated with involved airway distribution, time to diagnosis, and disease severity.

We aimed to identify distinct subgroups of tracheobronchial disease patterns in patients with AAV using LCA, and to evaluate identified subgroups in regards to clinical characteristics and outcomes.

## 2. Patients and Methods

We conducted a retrospective cohort study using electronic medical records from three institutional Mayo Clinic sites (Minnesota, Florida, and Arizona). Patients aged >18 years diagnosed with AAV, based on the revised 2012 International Chapel Hill Consensus Nomenclature, and diagnosed with tracheobronchial disease between 1 January 2002 and 6 September 2022, were included. The diagnosis was based on a combination of histopathology findings and the type of ANCA involvement. Patients who were difficult to classify based on the Chapel Hill Consensus Nomenclature were labeled as “unspecified ANCA disease”.

Evaluation for tracheobronchial disease could have been performed with laryngoscopy by otolaryngology or bronchoscopy with a pulmonologist. Patients with <6 months of follow-up were excluded. Clinical characteristics, pertinent serologic markers, and chest computed tomography (CT) findings were documented. Immunosuppressive regimens were documented along with the start date, end date, and reason for discontinuation. Immunosuppressive regimens included cyclophosphamide, rituximab, and methotrexate and these could be documented as induction agents or maintenance. Induction agents included rituximab or cyclophosphamide and were required to have been used within 6 months of the maintenance agent. Maintenance agents could include rituximab, methotrexate, mycophenolate, or azathioprine. Pulmonary function testing results included forced expiratory volume in the first second (FEV1) percent predicted and forced vital capacity (FVC), when available. Various demographic and clinical variables were collected and summarized as mean, standard deviation, median, interquartile range, and range for continuous variables, while categorical variables are summarized as count and percentage.

LCA was employed to identify disease subtypes using 10 pre-defined indicator variables: tracheal involvement, bronchial involvement, ANCA subtype (negative ANCA, PR3/C-ANCA, MPO/P-ANCA, or “other ANCA”), saddle nose deformity, presence of lung nodules and/or ground-glass opacities, symptoms in the eye, ear, or cardiovascular/gastrointestinal/nervous systems, renal involvement, and cutaneous involvement. Other ANCA was defined as any alternative combination of positive ANCA testing. Maximum likelihood estimation was performed with 10 repetitions per model. Models ranging from one to eight classes were estimated; models with more than eight classes were unstable and not considered further. Model selection was based on the AIC, with lower values indicating better fit. Additional fit and diagnostic criteria were assessed but did not influence the final model selection.

Patient and disease characteristics were summarized and compared across predicted classes using Kruskal–Wallis tests for continuous variables and Fisher’s exact tests for categorical variables. Time to recurrence was defined as the duration from the initiation of first-line medication (including methotrexate, azathioprine, rituximab, or cyclophosphamide) to the first confirmed recurrence of airway inflammation observed during laryngoscopy or bronchoscopy. Patients without recurrence were censored at their last follow-up. Survival curves were estimated using the Kaplan–Meier method, and differences in survival among latent classes were assessed using log-rank tests. Analysis was performed in R version 4.2.2. and SAS version 9.4. LCA analysis was performed using the poLCA package [18].

## 3. Results

**Figure 1:**
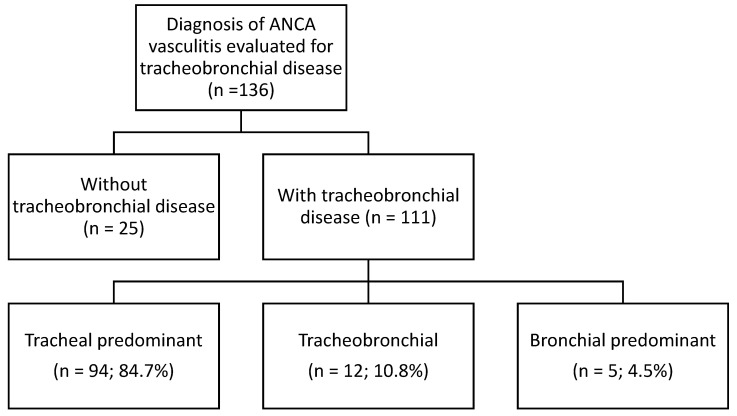
Flow chart of the study population.

A total of 136 patients with AAV who had undergone assessment for tracheobronchial involvement (Figure 1) with laryngoscopy or bronchoscopy were identified. After excluding 25 who did not have identified tracheal or bronchial disease, 111 were included.

### 3.1. Latent Classes Identified

A three-class model was deemed to be the best fit (Table 1). Table 2 identifies the baseline characteristics in each sub phenotype. The predicted probabilities of observing 10 variables per LCA classification are shown in Table 3. The first class identified, referred to as “tracheal predominant”, was a group of 94 patients with mostly subglottic involvement without bronchial inflammation and with less lower respiratory disease (Table 3). The second group “tracheobronchial” consisted of 12 patients with a higher percentage (50%) exhibiting concomitant tracheal disease with bronchial inflammation and lower respiratory disease. The third group “bronchial predominant” held five patients. These patients lacked subglottic involvement.

### 3.2. Baseline Characteristics by Predicted Class Assignment

Table 2 summarizes the demographic and clinical characteristics of the cohort. Average age was 41.0 years, 86 (77.5%) patients were female, 98 (88.3%) were White. The majority, 105 (94.6%) patients, held a diagnosis of GPA as opposed to MPA or unspecified ANCA vasculitis. A total of 96 (86.5%) patients were positive for any ANCA type, 106 (95.5%) of patients had tracheal stenosis, and 19 (17.1%) had bronchial stenosis. Age, sex, and ethnicity were similar between predicted classes (Table 4). The median time of follow-up was 8.1 years (IQR 3.6–13.4).

### 3.3. Immunosuppressive Management Within Cohort

Of the 111 patients, 44 (39.6%) utilized rituximab induction and maintenance in combination with another agent such as mycophenolate or methotrexate. Twenty-six (23.4%) patients utilized rituximab monotherapy as both induction and maintenance. Fifteen (13.5%) patients were treated with methotrexate without induction. Eight (7.2%) patients received induction therapy without maintenance. Of these 8 patients, 6 utilized rituximab, 1 utilized cyclophosphamide, and 1 was on a combination of rituximab and cyclophosphamide. Six (5.4%) patients were managed with azathioprine or mycophenolate without induction. Three (2.7%) patients utilized cyclophosphamide induction within 6 months of starting a maintenance agent (azathioprine, mycophenolate, or a combination). Four (3.6%) patients were not initiated on systemic immunosuppression. Three (2.7%) patients were maintained on an immunosuppressive agent not previously listed. Two (1.8%) patients did not have medication history available.

### 3.4. Probabilities of Disease Characteristics by Predicted Sub-Phenotype Classes

Class 1, tracheal predominant, was noted to have the highest prevalence of anti-MPO positivity (36.2%), though this was not statistically significant. Ear and eye involvement were less frequent than in other classes (16.0% and 7.4% prevalence, respectively). Lower respiratory involvement was less common (36.2%).

Class 2, tracheobronchial, had the highest prevalence of saddle nose deformity (50%) and lower respiratory (91.7%), renal (66.7%), cutaneous (41.7%), and other systemic manifestations (16.7%) out of all three classes. This severity with widespread disease was not linked to specific ANCA status.

Class 3, bronchial predominant, had frequent lower respiratory involvement (80%) and lacked saddle nose deformity, cutaneous disease, renal involvement, or other systemic ANCA manifestations.

### 3.5. Outcomes by Predicted Class

**Figure 2:**
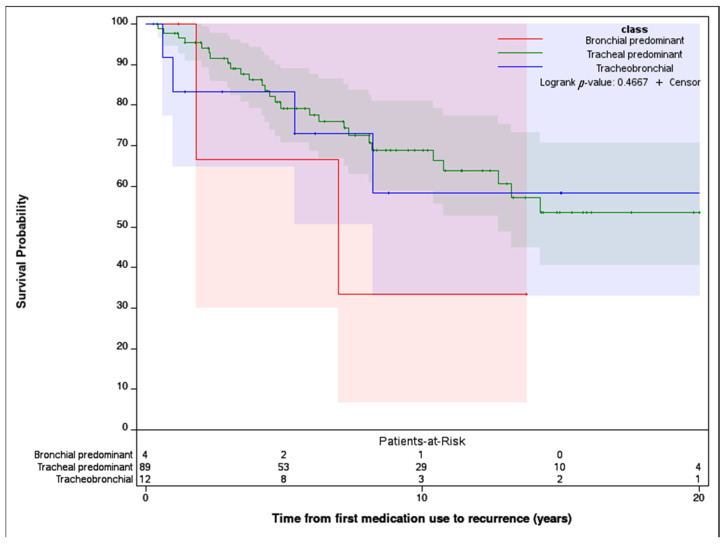
Relapse-free survival of tracheobronchial disease by predicted class. Shading represents the 95% confidence interval.

Time to recurrence of tracheobronchial inflammation after initiating immunosuppressive therapy, defined as the time from first medication start date to first recurrence (or loss of follow-up), was compared between classes (Figure 2) without significant difference. Secondary outcomes are shown in Table 4. Endoscopic intervention was common (59.5%) though rates did not differ significantly between classes. The infrequent occurrences of tracheostomy and death were not different between classes (6.3% and 1.8%, respectively).

In the 84 (75.7%) patients who had pulmonary function testing available it was noted that median FEV1 percent predicted decreased with class allocation (89.0, 75.0, and 58.0, respectively, with a *p* value of 0.24). The FEV1/FVC ratio was also statistically significant across classes 1, 2, and 3 (73.0, 67.0, and 56.9, respectively, with a *p* value of 0.31). 

## 4. Discussion

Tracheobronchial disease is a recognized consequence of ANCA-associated vasculitis, particularly GPA, and in this report, we have described a cohort of patients with AAV-associated tracheobronchial disease. Our findings align with previous reports, indicating that upper tracheal (subglottic) inflammation and stenosis are common [3,5,6,7,8,9,10,11,12,19,20].

As highlighted previously, there is a paucity of data regarding the epidemiology and outcomes of tracheobronchial disease in AAV with a limited number of studies available, and those that are available provide conflicting information [7,11,12,13,14]. The prevalence of tracheobronchial disease is felt to be around 20% but has been reported to be as high has 70% [8,12]. Previously, it has been reported that in patients with GPA, the subglottic stenosis variant of tracheobronchial disease may be more prevalent in younger and female patients with GPA and those with severe, destructive, sinonasal disease but a lack of renal disease [7]. Endobronchial disease was more prevalent in younger patients with positive PR3-ANCA, and those who has a history of ENT involvement. It remains unclear if the upper airway involvement is associated with a severe disease [7,8,10].

This analysis further builds on existing data and highlights the presence of sub-phenotypes within these cohorts. Patients with tracheal predominant disease were noted to have the highest prevalence of anti-MPO positivity and less common involvement of ear, eye, or lower respiratory tract. Tracheobronchial disease was associated with saddle nose deformity and lower respiratory, renal, cutaneous, and other systemic manifestations but not with specific ANCA status. The bronchial predominant subset had frequent lower respiratory involvement with obstructive lung disease, and lacked cutaneous, renal, or other systemic ANCA manifestations.

Most notably, systemic disease was seen in patients with more diffuse airway involvement, i.e., tracheobronchial disease, and less with isolated bronchial disease. These findings suggest that severe systemic presentation may herald a more severe upper airway involvement and vice-versa. The more frequent occurrence of renal disease in patients presenting with tracheobronchial disease suggests that this subset of patients should also be monitored closely for renal disease. Additionally, the degree of obstruction observed in pulmonary function tests was linked to both the tracheobronchial and bronchial predominant subgroups. Though the bronchial predominant group was small, there was an indication that those with isolated bronchial disease may have had less systemic manifestations.

It is important to note that this study observed less frequent involvement of the lower airways compared to earlier literature, with lower tracheal and bronchial disease each affecting less than 20% of the population. This discrepancy may be attributed to our study including patients who had undergone either laryngoscopy or bronchoscopy. Without bronchoscopy or chest computed tomography read by a skilled radiologist, the prevalence of lower airway involvement might have been underestimated.

Despite this limitation, LCA revealed three distinct subgroups with different phenotypic characteristics a useful construct that can be used during evaluation of these patients as well as for future research.

These innovative findings provide significant insight into the heterogeneity of the airway involvement in AAV and highlight the presence of three different phenotypic subgroups with prognostic and therapeutic implications. Clinicians can use these results to stratify the patients during evaluation to aggressively assess and closely follow for systemic disease and saddle nose deformities in patients with tracheobronchial disease, and potentially intervene early to improve the outcome. Furthermore, the findings provide a direction for additional studies in the future.

However, it is important to note that the phenotypic subgroups identified in this study did not show a correlation with outcomes outside of the degree of obstructive disease on pulmonary function testing, which seemed to be associated with bronchial airway involvement. Other outcomes, such as relapse rates of tracheobronchial disease following the initiation of immunosuppressive therapy or the necessity for endoscopic interventions like dilation and injection, did not correlate strongly. This lack of correlation is likely due to the small sample size, particularly in the bronchial predominant subgroup. Alternatively, this may also be due to a less aggressive use or the limited effectiveness of standard immunosuppressive therapies for tracheobronchial disease. The benefits of systemic immunosuppressive therapy for airway involvement are questionable based on current literature but there are instances of improved outcome with aggressive immunosuppressive therapy in addition to endoscopic or surgical interventions. Two recent studies show that immunosuppressive treatment, and in particular rituximab, may reduce the need for endoscopic interventions, supporting their use in AAV [15,16].

## 5. Conclusions

This LCA of patients with tracheobronchial disease and ANCA vasculitis identified distinct phenotypic subgroups, each characterized by unique disease features and association with distinct non-airway clinical manifestations. Despite these differences, our analysis did not reveal a significant association between these subgroups and clinical outcomes such as disease relapse or the need for endoscopic interventions. These findings suggest that while phenotypic heterogeneity exists, it may not directly translate into differences in clinical outcomes with the current treatment approaches. Future research should focus on further exploring the underlying mechanisms driving these subgroups and investigating whether more tailored or novel therapeutic strategies might improve outcomes for specific phenotypes. This could provide valuable insights for optimizing patient management and advancing personalized treatment in ANCA-associated tracheobronchial disease.

## Figures and Tables

**Table 1:** Model fit statistics for latent-class models from one to eight classes.

# of Classes	DF	AIC	BIC	Likelihood Ratio	Entropy
1	99	1173.376	1206	268.141	-
2	86	1160.303	1228	229.0679	1
3	73	1154.798	1258	197.5627	0.874
4	60	1166.587	1305	183.3519	0.81
5	47	1179.841	1353	170.606	0.779
6	34	1194.037	1403	158.802	0.816
7	21	1207.58	1451	146.3447	0.853
8	8	1221.306	1500	134.0711	0.879

Akaike Information Criterion (AIC) had the lowest value with the 3-class model indicating best fit. The entropy level for the 3-class model was also the highest at 0.874, suggesting that there was strong distinction between classes. This was coupled with a Bayesian information criterion (BIC) lower than that of models with higher number of classes, which suggests that further classification did not improve distinction between models. Additional abbreviations: DF, degrees of freedom.

**Table 2:** Baseline characteristics by predicted class assignment.

	Predicted Class				
Demographics	Tracheal Predominant (N = 94)	Tracheo-Bronchial (N = 12)	Bronchial Predominant (N = 5)	Total (N = 111)	*p*-Value
Age at vasculitis diagnosis					0.171 ^1^
Mean (SD)	42.3 (17.58)	32.8 (17.38)	35.8 (17.68)	41.0 (17.69)	
Median (IQR)	44.6 (26.5, 55.8)	27.0 (18.7, 46.9)	33.7 (25.1, 52.4)	43.6 (25.4, 55.1)	
Sex, n (%)					0.888 ^2^
Female	72 (76.6%)	10 (83.3%)	4 (80.0%)	86 (77.5%)	
Male	22 (23.4%)	2 (16.7%)	1 (20.0%)	25 (22.5%)	
Ethnicity, n (%)					1.000 ^2^
Caucasian	82 (87.2%)	11 (91.7%)	5 (100.0%)	98 (88.3%)	
Hispanic	7 (7.4%)	1 (8.3%)	0 (0.0%)	8 (7.2%)	
African American	2 (2.1%)	0 (0.0%)	0 (0.0%)	2 (1.8%)	
Native American	1 (1.1%)	0 (0.0%)	0 (0.0%)	1 (0.9%)	
Other	2 (2.1%)	0 (0.0%)	0 (0.0%)	2 (1.8%)	
Length of follow-up (weeks)					0.817 ^1^
mean (SD)	480.1 (314.22)	504.9 (473.81)	409.4 (341.21)	479.6 (332.34)	
Median (IQR)	437.1 (209.0, 680.0)	299.9 (171.0, 977.8)	310.6 (111.4, 687.0)	420.9 (186.4, 697.1)	
Disease Characteristics					
Tracheal stenosis, n (%)	94 (100.0%)	12 (100.0%)	0 (0.0%)	106 (95.5%)	<0.001 ^2^
Bronchial stenosis, n (%)	8 (8.5%)	6 (50.0%)	5 (100.0%)	19 (17.1%)	<0.001 ^2^
Diagnosis, n (%)					>0.999 ^2^
GPA	88 (93.6%)	12 (100.0%)	5 (100.0%)	105 (94.6%)	
MPA	4 (4.3%)	0 (0.0%)	0 (0.0%)	4 (3.6%)	
Unspecified ANCA-associated vasculitis	2 (2.1%)	0 (0.0%)	0 (0.0%)	2 (1.8%)	
ANCA, n (%)					0.098 ^2^
negative ANCA	13 (13.8%)	0 (0.0%)	2 (40.0%)	15 (13.5%)	
PR3/C-ANCA	37 (39.4%)	8 (66.7%)	3 (60.0%)	48 (43.2%)	
MPO/P-ANCA	34 (36.2%)	2 (16.7%)	0 (0.0%)	36 (32.4%)	
Atypical ANCA	10 (10.6%)	2 (16.7%)	0 (0.0%)	12 (10.8%)	
**Specific Organ Involvement**					
Cutaneous, n (%)	9 (9.6%)	5 (41.7%)	0 (0.0%)	14 (12.6%)	0.019 ^2^
Ear, n (%)	15 (16.0%)	12 (100.0%)	2 (40.0%)	29 (26.1%)	<0.001 ^2^
Eye, n (%)	7 (7.4%)	2 (16.7%)	2 (40.0%)	11 (9.9%)	0.042 ^2^
Saddle nose deformity, n (%)	13 (13.8%)	6 (50.0%)	0 (0.0%)	19 (17.1%)	0.011 ^2^
* Lower respiratory, n (%)	34 (36.2%)	11 (91.7%)	4 (80.0%)	49 (44.1%)	<0.001 ^2^
Renal, n (%)	14 (14.9%)	8 (66.7%)	0 (0.0%)	22 (19.8%)	<0.001 ^2^
** Systemic, n (%)	12 (12.8%)	2 (16.7%)	0 (0.0%)	14 (12.6%)	0.828 ^2^

^1^ Kruskal–Wallis *p*-value; ^2^ Fisher’s exact *p*-value; comparison of baseline characteristics by predicted class assignment. Abbreviations: SD, standard deviation; IQR, interquartile range; GPA, granulomatosis with polyangiitis; MPA, microscopic polyangiitis; ANCA, anti-neutrophil cytoplasmic antibody; PR3, proteinase-3; c-ANCA, cytoplasmic ANCA; MPO, myeloperoxidase; p-ANCA, perinuclear ANCA. *—indicates nodules, interstitial lung disease, or alveolar hemorrhage. **—indicates cardiac, nervous system, or renal involvement secondary to vasculitis.

**Table 3:** Probabilities of disease characteristics by predicted sub-phenotype classes.

Disease Characteristics	Bronchial Predominant	Tracheobronchial	Tracheal Predominant
Atypical ANCA	0.000	0.184	0.104
PR3	0.600	0.636	0.397
MPO	0.000	0.180	0.360
Negative ANCA	0.400	0.000	0.138
Subglottic involvement	0.000	1.000	1.000
Bronchial involvement	1.000	0.476	0.088
Saddle nose	0.000	0.458	0.143
Cutaneous	0.000	0.369	0.102
Ear	0.400	1.000	0.159
Eye	0.400	0.158	0.075
Lower respiratory involvement	0.800	0.911	0.362
Renal	0.000	0.618	0.155
Systemic	0.000	0.188	0.125

Class-conditional outcome probabilities were estimated from LCA modeling. Value in each cell is the probability of observing the disease characteristic for an individual in the predicted sub-phenotype. Cell color is determined by the value of the probability, with high probabilities given a darker shade and low probabilities given a lighter shade. Abbreviations: ANCA, anti-neutrophil cytoplasmic antibody; PR3, proteinase-3; MPO, myeloperoxidase.

**Table 4:** Outcomes by predicted class assignment.

	Predicted Class				
	Tracheal Predominant (N = 94)	Tracheo-Bronchial (N = 12)	Bronchial Predominant (N = 5)	Total (N = 111)	*p*-Value
**Pulmonary Function Tests**					
Available data, n (%) ****	72 (76.6%)	7 (58.3%)	5 (100.0%)	84 (75.7%)	0.201 ^2^
FEV1% predicted					0.024 ^1^
mean (SD)	86.8 (23.01)	77.1 (20.80)	55.4 (23.64)	84.0 (23.92)	
Median (IQR)	89.0 (73.0, 103.0)	75.0 (70.0, 95.0)	58.0 (34.0, 66.0)	88.0 (69.0, 102.0)	
FVC % predicted					0.505 ^1^
mean (SD)	97.4 (18.87)	96.9 (6.07)	85.2 (24.14)	96.6 (18.52)	
Median (IQR)	100.5 (84.5, 109.5)	98.0 (90.0, 101.0)	92.0 (76.0, 96.0)	99.0 (85.5, 108.5)	
FEV1/FVC ratio					0.031 ^1^
Mean (SD)	69.4 (13.48)	64.6 (15.86)	52.4 (17.12)	67.9 (14.37)	
Median (IQR)	73.0 (63.4, 78.3)	67.0 (58.7, 69.4)	56.9 (43.0, 63.3)	71.0 (60.4, 78.0)	
**Tracheobronchial Interventions**					
Endoscopic dilation, n (%)	54 (57.4%)	9 (75.0%)	3 (60.0%)	66 (59.5%)	0.497 ^2^
Number of dilations, n (%)					0.765 ^2^
1	21 (38.9%)	3 (33.3%)	2 (66.7%)	26 (39.4%)	
2	9 (16.7%)	2 (22.2%)	0 (0.0%)	11 (16.7%)	
3	9 (16.7%)	3 (33.3%)	0 (0.0%)	12 (18.2%)	
4	15 (27.8%)	1 (11.1%)	1 (33.3%)	17 (25.8%)	
Number of dilations					0.863 ^1^
Mean (SD)	2.3 (1.26)	2.2 (1.09)	2.0 (1.73)	2.3 (1.24)	
Median (IQR)	2.0 (1.0, 4.0)	2.0 (1.0, 3.0)	1.0 (1.0, 4.0)	2.0 (1.0, 4.0)	
Tracheostomy, n (%)	6 (6.4%)	1 (8.3%)	0 (0.0%)	7 (6.3%)	0.699 ^2^
**Mortality**					
Death during follow-up period, n (%)	2 (2.2%)	0 (0.0%)	0 (0.0%)	2 (1.9%)	1.000 ^2^
Missing	2	1	0	3	

^1^ Kruskal–Wallis *p*-value; ^2^ Fisher’s exact *p*-value; **** Among patients in the tracheal predominant group who had PFTs, 3 were missing FEV1% predicted, 4 were missing FVC% predicted, and 5 were missing FEV1/FVC ratio. Abbreviations: FEV1, forced expiration volume over one minute; SD, standard deviation; IQR, interquartile range; FVC, forced vital capacity.

## Data Availability

Data can be provided by the authors upon request, with removal of identifiable information and anonymization of dates.
